# Central nervous system manifestations of LRBA deficiency: case report of two siblings and literature review

**DOI:** 10.1186/s12887-023-04182-z

**Published:** 2023-07-13

**Authors:** T. C. Mangodt, K. Vanden Driessche, K. K. Norga, N. Moes, M. De Bruyne, F. Haerynck, V. Bordon, A. C. Jansen, A. I. Jonckheere

**Affiliations:** 1 Division of Pediatric Neurology, Department of Pediatrics, Antwerp University Hospital, Drie Eikenstraat 655, 2650 Edegem, Belgium; 2 Pediatric Infectious Diseases, Department of Pediatrics, Antwerp University Hospital, Edegem, Belgium; 3 Division of Pediatric Hematology-Oncology, Department of Pediatrics, Antwerp University Hospital, Edegem, Belgium; 4 Division of Pediatric Gastro-Enterology, Department of Pediatrics, Antwerp University Hospital, Edegem, Belgium; 5 Center for Medical Genetics Ghent, Ghent University Hospital, Ghent, Belgium; 6 Department of Biomolecular Medicine, Ghent University Hospital, Ghent, Belgium; 7 Department of Pediatric Immunology and Pulmonology, Ghent University Hospital, Ghent, Belgium; 8 Department of Pediatric Hematology-Oncology and Stem Cell Transplantation, Ghent University Hospital, Ghent, Belgium

**Keywords:** Central nervous system, LRBA deficiency, Neurological, Hearing loss, Case report, MRI

## Abstract

**Background:**

LPS-responsive beige-like anchor protein (LRBA) deficiency is a primary immunodeficiency disease (PID) characterized by a regulatory T cell defect resulting in immune dysregulation and autoimmunity. We present two siblings born to consanguineous parents of North African descent with LRBA deficiency and central nervous system (CNS) manifestations. As no concise overview of these manifestations is available in literature, we compared our patient’s presentation with a reviewed synthesis of the available literature.

**Case presentations:**

The younger brother presented with enteropathy at age 1.5 years, and subsequently developed Evans syndrome and diabetes mellitus. These autoimmune manifestations led to the genetic diagnosis of LRBA deficiency through whole exome sequencing with PID gene panel. At 11 years old, he had two tonic–clonic seizures. Brain MRI showed multiple FLAIR-hyperintense lesions and a T2-hyperintense lesion of the cervical medulla.

His sister presented with immune cytopenia at age 9 years, and developed diffuse lymphadenopathy and interstitial lung disease. Genetic testing confirmed the same mutation as her brother. At age 13 years, a brain MRI showed multiple T2-FLAIR-hyperintense lesions. She received an allogeneic hematopoietic stem cell transplantation (allo-HSCT) 3 months later. Follow-up MRI showed regression of these lesions.

**Conclusions:**

Neurological disease is documented in up to 25% of patients with LRBA deficiency. Manifestations range from cerebral granulomas to acute disseminating encephalomyelitis, but detailed descriptions of neurological and imaging phenotypes are lacking. LRBA deficiency amongst other PIDs should be part of the differential diagnosis in patients with inflammatory brain lesions. We strongly advocate for a more detailed description of CNS manifestations in patients with LRBA deficiency, when possible with MR imaging. This will aid clinical decision concerning both anti-infectious and anti-inflammatory therapy and in considering the indication for allo-HSCT.

**Supplementary Information:**

The online version contains supplementary material available at 10.1186/s12887-023-04182-z.

## Background

**Fig. 1:**
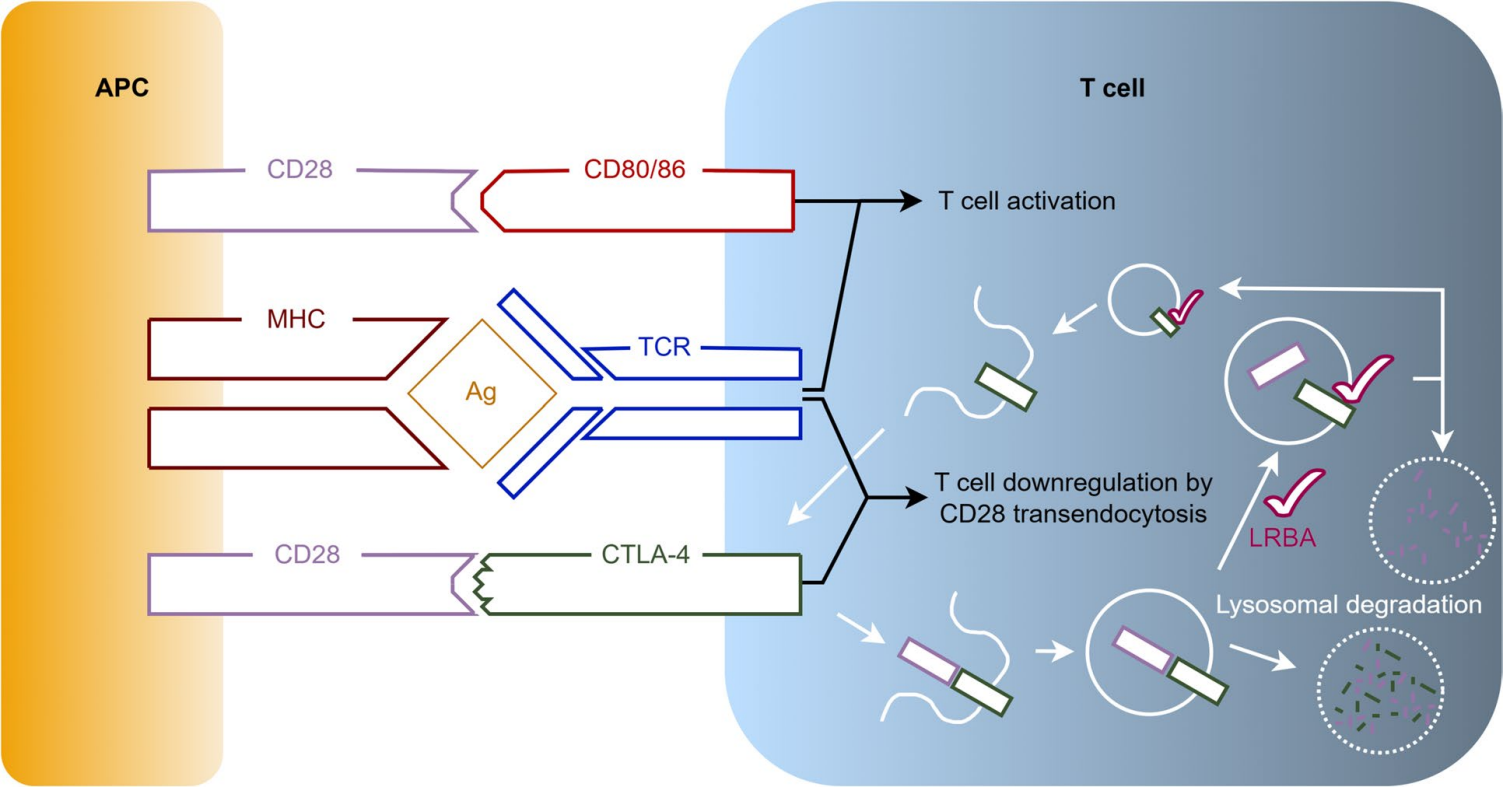
Overview of the role of LRBA in the interaction of antigen-presenting cells and T cells. To activate a T cell, an APC couples both its MHC with the TCR, and its CD28 cell surface molecule with the T cell's CD80/86 cell surface molecule. After activation of the TCR, CTLA-4 is trafficked to the T cell’s surface where it can also bind the APC’s CD28 molecule. In contrast to CD80/86, CTLA-4 downregulates the T cell’s activation by removing the CTLA-4/CD28-complexes from the cell surface. Once internalised, this complex is either degraded in lysosomes, or the CTLA-4 component is recycled thanks to the presence of LRBA and returned to the T cell’s surface allowing further downregulation. Abbreviations used: Ag: Antigen; APC: Antigen-presenting Cell; CD: Cluster of Differentiation; CTLA-4: Cytotoxic T lymphocyte Antigen 4; LRBA: Lipopolysaccharide-responsive Beige-like Anchor Protein; MHC: Major Histocompatibility Complex; TCR: T cell Receptor

Lipopolysaccharide-responsive beige-like anchor protein (LRBA) deficiency is a primary immunodeficiency disease (PID) caused by biallelic mutations in the *LRBA* gene. This gene is located on 4q31.3 and encodes the LRBA protein containing 2851 amino acids. The LRBA protein is expressed in many cell types, including hematopoietic, gastrointestinal, endocrine and neural cells [1]. Its highest expression, however, is found in immune cells [2]. The LRBA protein contains a WD40 repeat domain, and thereby functions as a protein interaction scaffold [3]. As such, its role in vesicle trafficking and signal transduction is especially important for the immune defense against bacterial lipopolysaccharides (LPS), the major outer surface membrane components present in almost all Gram-negative bacteria [4]. As demonstrated in Fig. 1, an antigen-presenting cell activates a T cell by coupling its Major Histocompatibility Complex (MHC) with the T cell receptor (TCR). To achieve full T cell activation, a costimulatory interaction is required between the APC’s Cluster of Differentiation 28 (CD28) cell surface molecule and the T cell’s CD80/86 cell surface molecule. After activation of the TCR, cytotoxic T lymphocyte antigen 4 (CTLA-4) is trafficked to be expressed on the T cell’s surface where it can also bind to the APC’s CD28 molecule. However, as opposed to CD80/86, CTLA-4 downregulates the T cell’s activation by removing the CTLA-4/CD28-complexes from the cell surfaces through transendocytosis. Once internalised, this complex is either degraded in lysosomes, or the CTLA-4 component is recycled and returned to the T cell’s surface. The latter requires the presence of LRBA in the endosomes. In the case of LRBA-deficiency, the only route CTLA-4 is able to follow is that of lysosomal degradation, thus reducing the CTLA-4 protein levels to 50–75% in most cases [5]. While conventional T cells require TCR activation to express CTLA-4 on the cell surface, it is constitutively present on regulatory T cells (Treg) [6]. As Treg cells are essential in controlling T cell proliferation and differentiation to maintain immune homeostasis and self-tolerance, LRBA deficiency hampers their ability to do so [7]. The reduced levels of the downregulating CTLA-4 molecule on the T cell’s surface and the dysfunctional controlling Treg cells result in an aberrantly prolonged T cell activation with multi-organ lymphocytic infiltration and high numbers of auto-reactive lymphocytes, all leading to auto-immunity [5].

These features of auto-immunity are the backbone of the clinical picture associated with LRBA deficiency. Onset is generally in the first two years of life. Most frequently, patients suffer from hematologic auto-immune disorders such as auto-immune hemolytic anemia (AIHA) and immune thrombocytopenic purpura (ITP). Many suffer from chronic diarrhea due to auto-immune enteropathy. Auto-immune endocrinopathies such as diabetes mellitus and auto-immune thyroiditis are common as well [8]. Most patients also suffer from hypogammaglobulinemia, which can be explained by gradual B cell exhaustion caused by overstimulation and/or infiltration of the bone marrow by T cells [5]. The immune dysregulation characteristic in these patients leads to infections, most commonly of the airways (pneumonia, sinusitis, otitis media) and gastro-intestinal tracts. Lymphoproliferative disorders are common (mainly organomegaly and lymphadenopathy), and granulomatous lesions can form in many organs [8]. While rare in common variable immunodeficiency disorders (CVID), LRBA deficiency is frequently associated with granulomatous lung disease as well as granulomatous lymphocytic interstitial lung diseases (GLILD) [9]. Neurological disease has been described in up to 25% of patients, mainly as a direct result of autoinflammatory disease, with occurrence of cerebral granulomatous lesions, nerve demyelination and atrophy [1]. Indirectly, neurological disease can also result from immune thrombocytopenic purpura leading to cerebral hemorrhages [10–12].

Diagnosis of LRBA deficiency is based on the demonstration of biallelic pathogenic genetic variants in the *LRBA* gene. These can be found throughout the whole gene (there is no mutational hotspot), and are mostly frameshift (insertion/deletion), nonsense or missense mutations [6]. There is no genotype–phenotype correlation in LRBA deficiency [1, 9, 13], with the exception of enteropathy [8] and polyautoimmunity [6] in case of severe mutations (nonsense or indel as opposed to missense or splice-site mutations). Additionally, LRBA proteins can be measured with flow cytometry or western blotting (WB), but care needs to be taken since some *LRBA* gene mutations can result in residual expression or genetic reversion mosaicism [8, 9].

Treatment is based on immunosuppression, for which sirolimus and abatacept are now favored above more conventional treatment modalities (corticosteroids, rituximab, azathioprine and others) [14, 15]. Recent findings support allogenic hematopoietic stem cell transplantation (allo-HSCT) for patients with severe presentations of LRBA deficiency. Long-term survival probability remained comparable between patients with or without allo-HSCT, but the allo-HSCT survivors showed stable remission of LRBA deficiency-related symptoms and most did not require further immunosuppression. In contrast, those receiving conventional treatment experienced higher disease burden throughout their lives and required more medication overall. As LRBA deficiency is a lifelong disease with risk of malignancy, the expected amelioration of disease manifestations must be individually weighed against the risks involved with allo-HSCT [14].

This report presents two siblings born to consanguineous parents of North African origin with LRBA deficiency who presented central nervous system (CNS) manifestations, and we compared our findings with the available literature.

## Case presentations

### Patient 1

**Fig. 2:**
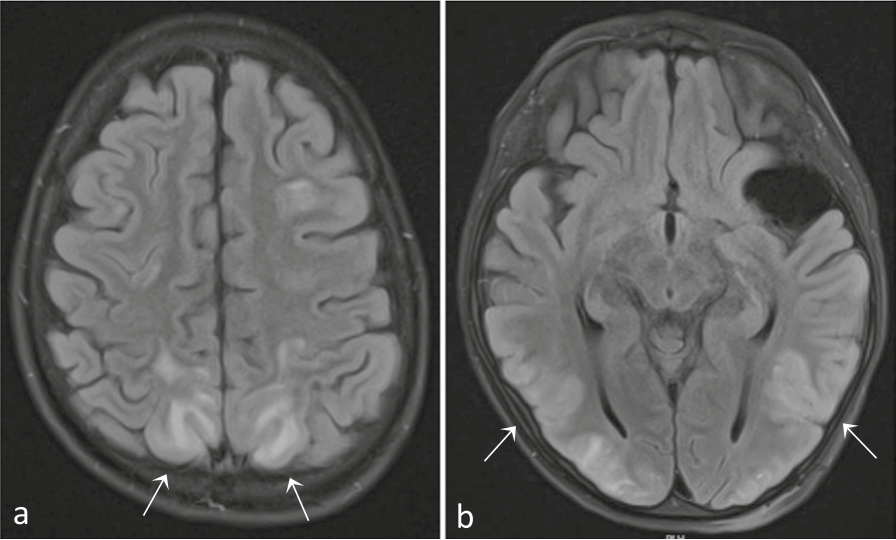
T2-Fluid-attenuated inversion recovery (FLAIR) images (**a** and **b**) demonstrating bilateral parieto-occipital hyperintense lesions. Images in conjunction with those in Supplemental figure S1 compatible with PRES. As an incidental finding, **a** subarachnoidal cyst in the left fossa media can also be observed (**b**). Images from patient P1

Patient 1 (P1) is a boy who presented at the age of 1.5 years with chronic diarrhea and failure to thrive due to a protein-losing enteropathy. At the age of 3.5 years he developed pancytopenia and was diagnosed with Evans syndrome (auto-immune hemolytic anemia and immune thrombocytopenia). There was a good treatment response to prednisone, which was given for two months. In the next half year, new episodes of immune cytopenia occurred: the first responded to intravenous immunoglobulins (IVIG), the second was resistant to IVIG but responsive to ciclosporin, and the third necessitated adding prednisolone to the treatment. Since then, P1 was free of recurrences for almost a year. At 5 years old he required higher doses of ciclosporin because of yet another episode of immune thrombocytopenia, which was complicated by transient renal failure and a posterior reversible encephalopathy syndrome (PRES; Fig. 2 and Fig. S1). Ciclosporin was discontinued and replaced by tacrolimus, but after four doses it was withdrawn due to the renal failure as well. Rituximab was initiated with good effect. Maintenance therapy with low-dose prednisolone was continued, and repeated IVIG substitution therapy was necessary due to hypogammaglobulinemia after rituximab. Half a year later, a new magnetic resonance imaging (MRI) scan of the brain showed complete remission of the PRES-lesions.

P1 was again in good health for another year until the age of 6 years, when he developed a new episode of immune thrombocytopenia that resolved successfully with a dose of rituximab. Half a year later, a new episode of auto-immune enteropathy necessitated restarting treatment with sirolimus, again with good response. At the age of 7 years, he was diagnosed with diabetes mellitus. Antibody titers (mainly glutamic acid decarboxylase antibodies (GADA), positive at 56U/ml) were indicative of an auto-immune etiology. He remained free of auto-immune crises for most of his primary school period. At the age of 9.5 years the final genetic diagnosis of a LRBA deficiency was made, triggered by a hospitalisation of his sister (P2). Genome-wide homozygosity mapping was performed, which indicated significant homozygous regions (> 1 Mb) in which, among other genes, the *LRBA* gene was located. Upon repeated attempts with homozygosity-guided targeted next-generation sequencing (Illumina sequencing by synthesis technology) and subsequent Sanger sequencing, exon 43 of the *LRBA* gene could not be amplified, suggestive of a homozygous deletion. A possible deletion of chr4:150,487,732–150,487,834 spanning exon 43 of the *LRBA* gene was confirmed using the ExomeDepth algorithm, a copy-number variant tool for exome data..

**Fig. 3:**
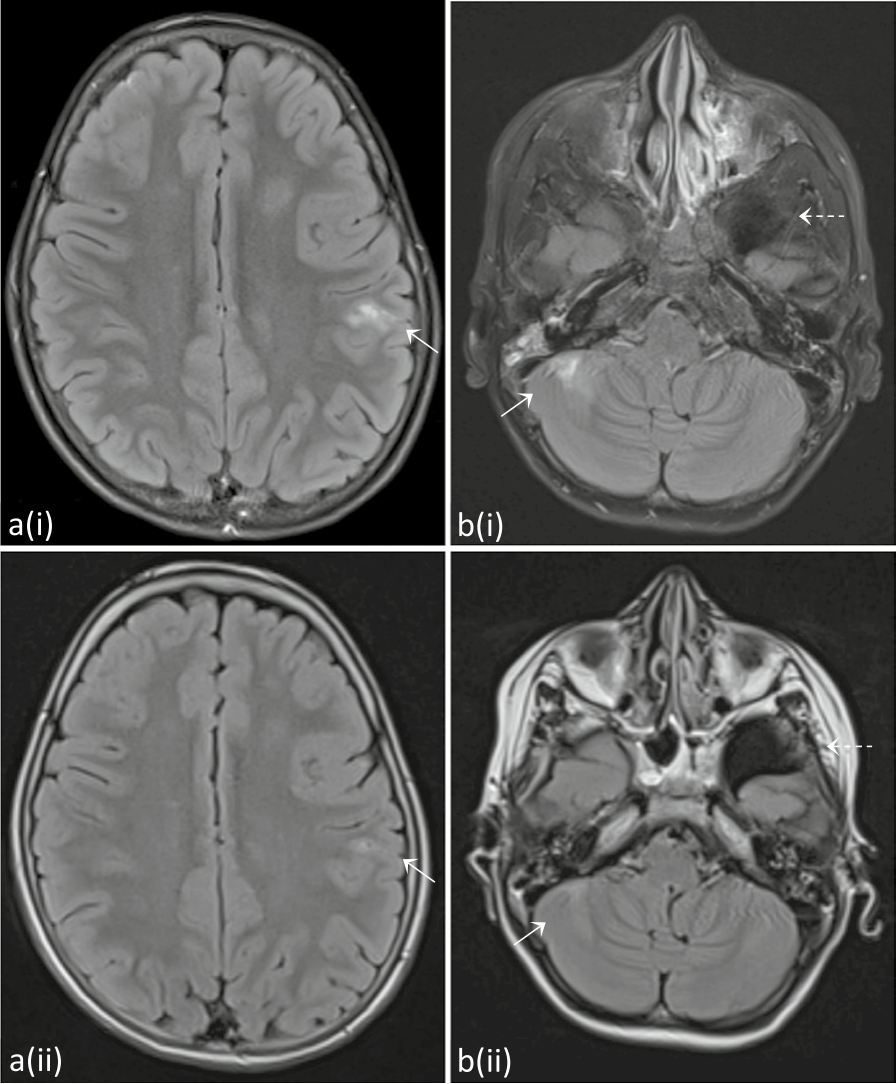
T2-FLAIR hyperintense contrast-enhanced lesions occurring widely spread in the supratentorial (**a**) and infratentorial (**b**) grey matter. As in Fig. 2, the arachnoidal cyst in the left temporal region can also be observed (b, dashed arrow). Evolution after 6 months of treatment with abatacept can be seen on the follow-up images depicted with (ii), demonstrating global regression of the lesions and decreased contrast enhancement. Images from patient P1

**Fig. 4:**
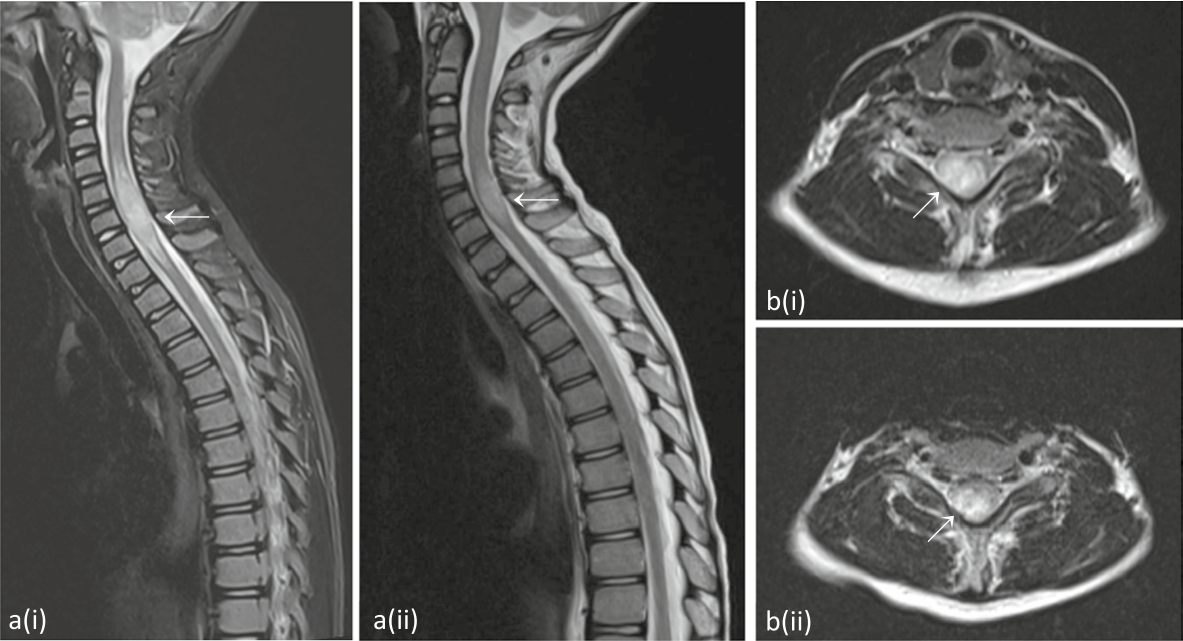
Expansive lesion of the cervical spinal medulla from C3-C4 down to Th1-Th2, best appreciated on the T2-weighted short-tau inversion recovery (STIR) image (**a**) and the T2-weighted image (**b**). Evolution after 6 months of treatment with abatacept can be observed on the follow-up images depicted with (ii), demonstrating reduced extent. Please note that image a(ii) is a T2-weighted image without STIR. Images from patient P1

When P1 was 10.5 years old, he presented at the emergency department after a first generalized tonic–clonic seizure that ceased after administration of diazepam. At the age of 11 years, a new seizure with focal-to-bilateral tonic–clonic semiology occurred for which he required multiple doses of midazolam. Work-up with electroencephalogram (EEG), fundoscopic examination and computed tomography (CT) scan were all normal. Consecutive lumbar punctures performed after the second seizure demonstrated slightly elevated leukocytes (7 and 20/µl), but no growth on cultures, negative viral and bacterial PCR testing and absence of oligoclonal bands. A brain MRI was performed, and demonstrated multiple FLAIR-hyperintense contrast-enhanced lesions in the supra- and infratentorial grey matter (Fig. 3 and Fig. S2). An additional spine MRI revealed a large T2-hyperintense cervical lesion with patchy contrast enhancement (Fig. 4 and Fig. S3). There were no associated neurological signs, in particular no lower limb motor impact, no dysautonomia and no urinary or fecal retention. This prompted further analysis on the obtained cerebrospinal fluid, which was negative for malignancy, and only demonstrated an elevated amount of reactive lymphocytes. Auto-immune antibodies were all negative. As part of further work-up, a lung CT revealed multiple micronodules as well. Voriconazole was temporarily given to cover the differential diagnosis of an opportunistic fungal infection until the results of the biopsy of one of the lung nodules came back negative.

As P1 had multisystem involvement (auto-immune diabetes mellitus, auto-immune enteropathy, Evans syndrome, CNS and lung lesions), treatment was initiated with abatacept, a CTLA-4-immunoglobulin fusion protein [16]. Levetiracetam was started and the patient has been free from seizures ever since. After 6 months of treatment with abatacept, at the age of 11.5 years, follow-up imaging of the CNS demonstrated global regression (but not resolution) of all lesions (Figs. 3, 4, S2 and S3). There were no new lesions. Currently, P1 is being considered for allo-HSCT. In preparatory work-up, a sensorineural hearing loss was discovered (while he had no subjective auditory problems) with tonal audiometry demonstrating a mixed low-frequency and high-frequency loss of 35 dB on the right and 55 dB on the left ear.

### Patient 2

Patient 2 (P2) is the older sister of P1. She presented at the age of 9 years with immune thrombocytopenia and a positive direct Coombs test. Her thrombocytopenia was resistant to IVIG but responsive to prednisolone. Because of recurrent episodes of immune thrombocytopenia, rituximab was added at the age of 11 years. One year later, she presented with abdominal pain and mildly elevated inflammatory parameters. Abdominal ultrasound demonstrated splenomegaly, an infectious etiology was suspected and treatment with amoxicillin-clavulanic acid successfully resolved all her symptoms, but not the splenomegaly. At the age of 12 years old, she was hospitalised with an interstitial lung disease with diffuse lymphadenopathies. No infectious pathogens were found despite of multiple investigations, including anatomopathological assessment of bronchoalveolar lavage fluid and a transbronchial needle aspiration of a paratracheal lymph node. Treatment with high-dose corticosteroids was initiated and slowly tapered over the next year. Due to this severe presentation requiring lengthy hospitalisation, as described above for P1, genetic analysis resulted in the diagnosis of a LRBA deficiency due to a deletion of chr4:150,487,732–150,487,834. This prompted additional treatment with sirolimus and after a few months abatacept was added as well. Follow-up CT imaging of the lungs demonstrated regression of the lung lesions, and her lung capacity increased from 35% to more than 90% over the course of a year. It was concluded that the lung lesions were part of the GLILD seen in patients with PID.

**Fig. 5:**
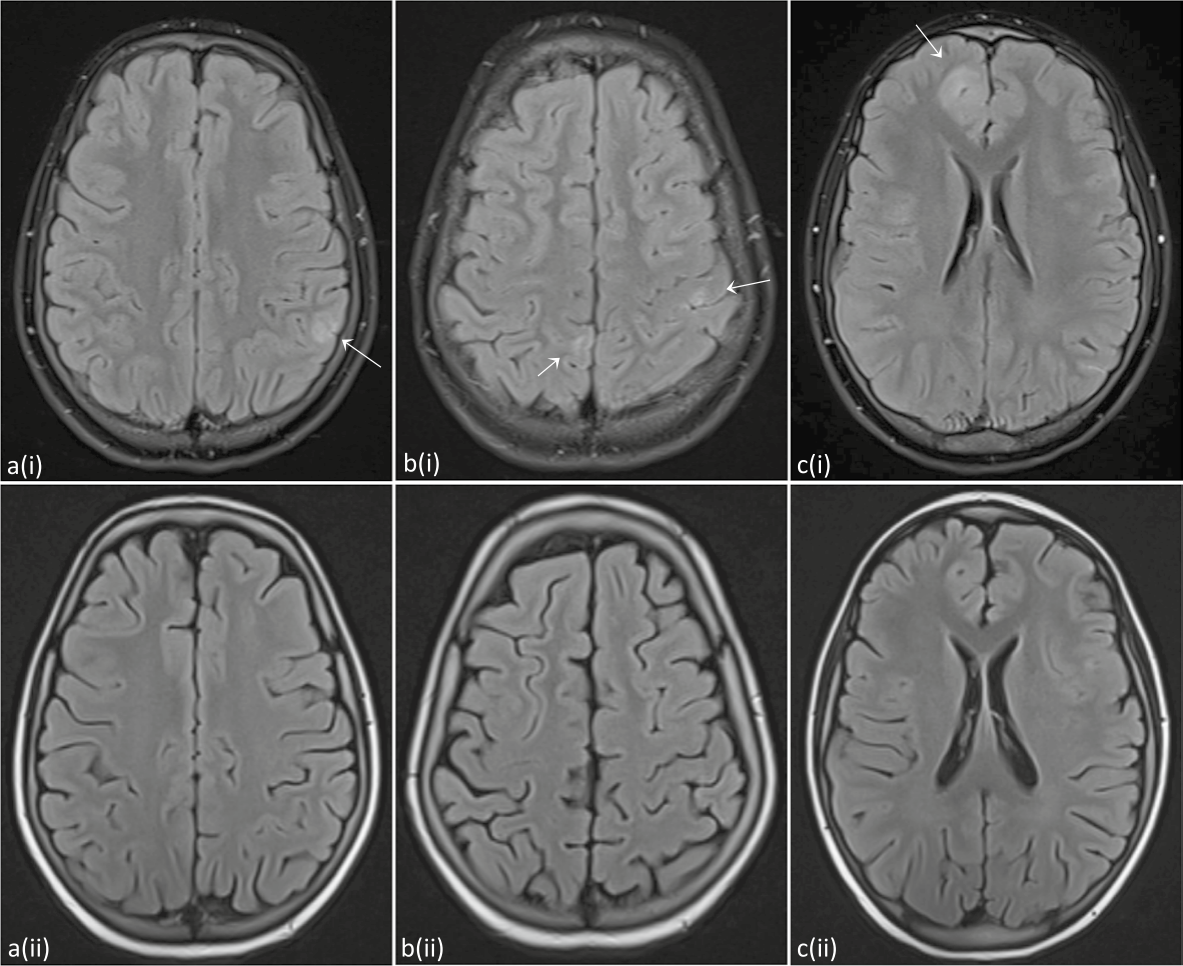
T2-FLAIR hyperintense subcortical supratentorial lesions with rather patchy contrast enhancement. Evolution three months after allo-HSCT can be observed on the follow-up images depicted with (ii), demonstrating full resolution of the lesions. Images from patient P2

At the age of 13 years she had new pulmonary complaints and a CT scan of the lungs demonstrated an increase in nodular lesions. An Aspergillus antigen test returned positive. Treatment with voriconazole was initiated. Because of headache and irritability with a normal clinical exam, central imaging was performed. Brain MRI showed multiple T2-FLAIR-hyperintense cortical to subcortical lesions with partial patchy contrast enhancement (Fig. 5). A lumbar puncture demonstrated pleocytosis (53/µl), but no other abnormalities (including cultures, PCR’s and flowcytometry). Follow-up brain MRI two months later demonstrated a fluctuating evolution of the lesions, some increased and some decreased.

Since P2 developed polyautoimmunity with multi-organ involvement (immune thrombocytopenia, splenomegaly, GLILD, CNS lesions) she received an allo-HSCT at the age of 14 years. The donor was a HLA-identical sister, heterozygous carrier of the *LRBA* gene deletion. In pre-transplant work-up a sensorineural hearing loss was discovered (as she too, did not suffer from subjective auditory problems): tonal audiometry demonstrated a severe high-frequency hearing loss of 95 dB on the right ear and 80 dB on the left. One year after transplantation a donor-chimerism of 89–91% was found.

**Fig. 6:**
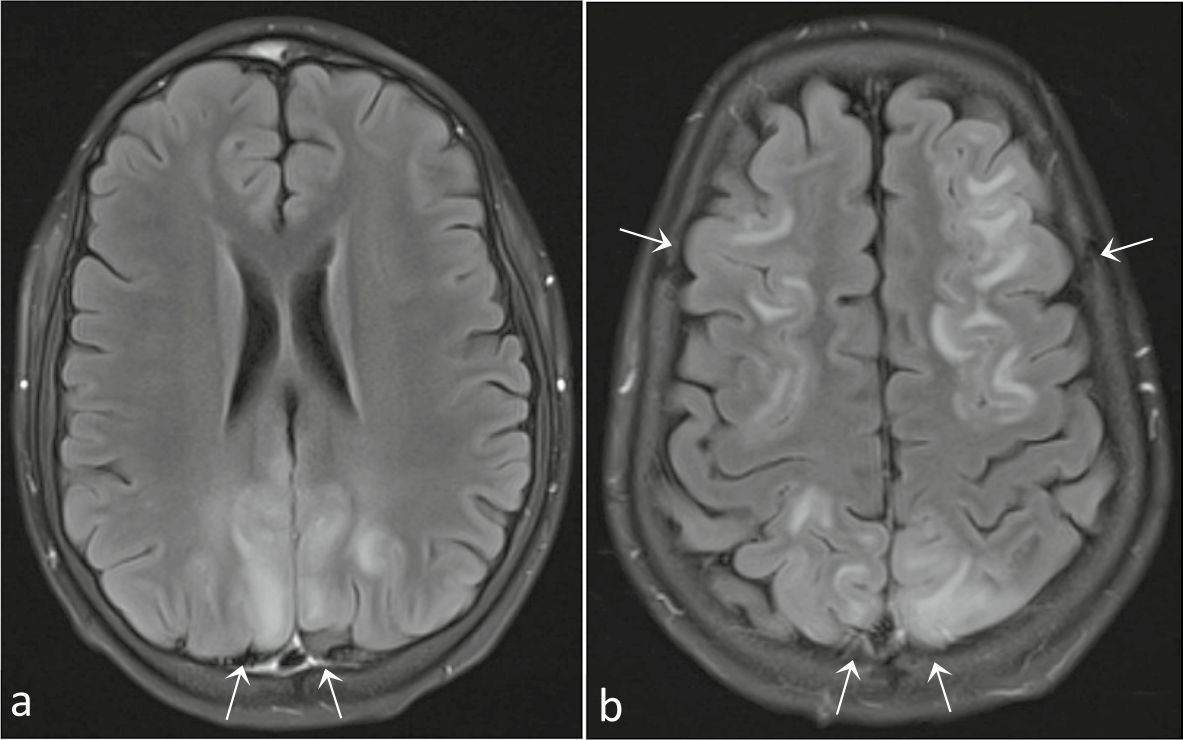
T2-FLAIR images (**a** and **b**) demonstrating bilateral fronto-parieto-occipital hyperintense lesions. Images in conjunction with those in Supplemental figure S4 compatible with PRES. Images from patient P2

Follow-up MRI of the brain three months after the allo-HSCT confirmed full resolution of all reported brain lesions, and an additional MRI of the spinal cord was normal. A CT scan of the lungs documented mainly a decrease of nodular lesions, but a few new nodular lesions had appeared. Four months after her allo-HSCT she developed a new auto-immune hemolytic crisis with splenomegaly and a CT scan of the lungs revealed further increase of granulomatous lung lesions. Treatment with prednisolone and sirolimus was initiated, the latter of which the doses had to be gradually reduced due to toxic concentrations (up to 22.3 µg/l). This resulted in quick recovery of her blood cell lines and a decrease of the lung nodules on follow-up CT scans of the lungs. After two weeks of treatment P2 presented at the emergency department with generalized tonic–clonic seizures. Lumbar puncture was not contributive, but an MRI revealed hallmark signs of a PRES (Fig. 6 and Fig. S4). Doses of sirolimus were further reduced, and a follow-up MRI after almost two months demonstrated resolution of the lesions.

Hitherto, P2 is stable under immunosuppressive treatment, but required addition of isavuconazole for Aspergillosis lesions in her lungs and addition of mycophenolate mofetil due to an upsurge of auto-immune cytopenia at the age of 14.5 years.

## Discussion and conclusions

We presented two siblings with LRBA deficiency. P1 developed auto-immune enteropathy, immune cytopenia, diabetes mellitus and CNS and lung lesions. Treatment with abatacept was beneficial in regressing all lesions. P2 developed immune thrombocytopenia, splenomegaly, GLILD and CNS lesions. Treatment with allo-HSCT resulted in full resolution of the lesions. We performed a review of the literature in PubMed to compare our central nervous system findings to those previously documented. Neurological disease has been described in 12 [6] – 25% of LRBA deficiency patients [1, 14, 17]. These manifestations mainly consist of demyelinating syndromes and cerebral granulomas, as summarized in Table 1. In two patients biopsies are available of the cerebral granulomas, and these demonstrated aspecific but prominently lymphocytic inflammatory infiltrative changes [1, 2]. In addition, CNS malignancies were reported in two patients: an astrocytic tumor and a CNS lymphoma [14], but these were not included in the table due to unavailability of case details.

**Table 1:** Reported neurological manifestations in LRBA deficiency

Manifestation	Ethnicity	Age of onset of LRBA deficiency symptoms	Age of neurological manifestation	Consanguinity	Sex	Ref	Ref. patient	Also referenced as
*Neuroinflammatory disease of the CNS*								
Cerebral granuloma with strabismus and hemiplegia	Arabian	1y	Young adult (± 20y)	Yes	M	[2]	P1	[1] (P1)
Cerebral granuloma with seizures^a^	Sicilian	12y	26y	Yes	M	[2]	P3	[1] (P3)
Cerebral granuloma-like lesion with unilateral optic nerve demyelination and atrophy^b^	Iranian	5y	12y	Yes	F	[1]	P31	[17] (P9), [13] (P2)
Cerebral granuloma-like lesions (multiple frontal)	-	7y	19y	-	M	[18]	P6	-
Parietal lobe lesion with seizures	Lebanese	3y	4y	Yes	F	[1]	P29	-
Multiple scattered focal bilateral T2/FLAIR hyperintense lesions in cortex and white matter	Caucasian	3y	6y	No	F	[19]	Index patient	-
Cerebral and cerebellar atrophy	Turkish	6 m	13y	Yes	F	[20]	Index patient	[11] (P1), [1] (P14)
Optic nerve atrophy, unilateral	Iranian	2y	5y	Yes	M	[1]	P28	[17] (P7)
MS (Optic neuritis and enhancing plaque on the corpus callosum)	-	1y	-	Yes	F	[17]	P12	[15] (N2)
Optic neuritis with demyelinating disease and multiple plaques in the brain	-	3.5y	> 17y	Yes	M	[16]	P12	-
Facial nerve palsy (unilateral, right-sided)	Turkish	7y	13y	Yes	M	[21]	Index patient	[22] (P1), [14] (P18)
ADEM			14y					
LETM	Italian	6 m	5y	No	F	[10]	Index patient	-
*Seizures*								
Seizures, no further details nor CNS imaging	-	6 m	-	Yes	M	[18]	P9	-
Seizures, no further details nor CNS imaging	Egyptian	6y	-	-	M	[23]	P10	-
Seizures, no further details nor CNS imaging	Egyptian	4 m	-	-	M	[23]	P11	-
Seizures, no further details nor CNS imaging	Egyptian	9 m	-	Yes	M	[12]	P6	-
*Neuromuscular disease*								
Demyelinating polyneuropathy	Turkish	6 m	13y	Yes	F	[20]	Index patient	[11] (P1), [1] (P14)
Myasthenia gravis	Iranian	2y	17y?	Yes	F	[2]	P5	[24] (P3), [1] (P5), [17] (P16), [15] (N15)
*Hearing loss*								
Sensorineural hearing loss	Sicilian	12y	26y	Yes	M	[2]	P3	[1] (P3), [25] (P1)
Sensorineural hearing loss	Caucasian (German)	4y	11y	No	F	[9]	P1 (105–1)	[25] (P2)
Sensorineural hearing loss	Egyptian	9 m	-		M	[23]	P6	-
Sensorineural hearing loss	-	12y	-	-	F	[18]	P4	-
*Cerebrovascular disease*								
Cerebral hemorrhage due to ITP	Turkish	7w	4y	No	M	[12]	P1	-
Cerebral hemorrhage due to ITP	Asian (Japan)	9y	15y	No	M	[9]	P11 (553–2)	-
Cerebral hemorrhage due to ITP	Turkish	Neonatal	4y	No	M	[11]	P3	-
Multiple cerebral infarctions	Egyptian	9 m	-	Yes	M	[12]	P6	-
*Intracranial hypertension*								
Pseudotumor cerebri	-	13y	-	Yes	F	[26]	P14	-

Abbreviations used: *ADEM *Acute Disseminated Encephalomyelitis, *CNS *Central Nervous System, *F *Female, *ITP *Immune Thrombocytopenic Purpura, *LETM *Longitudinally Extensive Transverse Myelitis, *m *months, *M *Male, *MS *Multiple Sclerosis, *w *weeks, *y *years

^a^On biopsy: granulomatous infiltration with T cells, plasma cells, and macrophages but low B cell numbers.

^b^On biopsy: mixed (non-malignant) lymphohistiocytic infiltrates and poorly formed granulomas, with predominantly CD3 + lymphocytes

Unfortunately, despite the fact that several articles mentioned central nervous system involvement [1, 2, 10, 16–21], only 4 provided MR images [10, 18, 19, 21]. Those provided by Tesi et al*.* and Semo Oz et al*.* share imaging characteristics with our patients [19, 21], namely scattered areas of focal T2/FLAIR hyperintensity in both cortical and subcortical regions with contrast enhancement. As LRBA deficiency is a rare auto-immune disease (< 1/1 000 000), few (paediatric) neurologists and radiologists are acquainted with the imaging characteristics. Treatment consists of immunosuppressive medication, increasing the risk for (opportunistic) central infectious diseases. The majority of patients with LRBA deficiency (74,5%) have polyautoimmunity [15], leading to polypharmacy and an increased risk of drug-induced phenomena (such as the PRES in both of our patients). These factors impede the distinction between opportunistic infections, drug-related CNS adverse events and intrinsic disease progression when central nervous system lesions are seen on brain or spinal cord imaging in patients with LRBA deficiency. Accurate diagnosis of these imaging abnormalities has therapeutic consequences, as neurological manifestations are an important parameter in determining the indication for allo-HSCT. As such, neurological involvement is also embodied as an independent parameter that increases the Immune Deficiency and Dysregulation Activity (IDDA) score, meant to objectivate disease burden in LRBA deficiency patients and used to guide clinical decision-making [14].

Both patients developed a PRES in their years of follow-up. In P1, two weeks before diagnosis of the PRES, ciclosporin was ceased after development of acute kidney failure with a serum peak concentration of 1770 µg/l and trough concentration of 207 µg/l (normal range ​600–1700 µg/l). Three days before diagnosis of the PRES, P1 also received tacrolimus for some days leading to a trough concentration of 15,9 µg/l, which is too high (normal range 5-15 µg/l). P2 developed PRES two weeks after initiation of sirolimus, which also had excessive trough concentrations up to 22,3 µg/l (normal range 4-20 µg/l). Despite multiple risk factors for PRES present in both patients (immunosuppressive treatment, underlying auto-immunity, renal failure, allo-HSCT in P2 [27]) it remains notable that P1 developed the PRES after contact with toxic concentrations of two calcineurin inhibitors (ciclosporin and tacrolimus) and P2 after toxic concentrations of the mammalian target of rapamycin (mTOR) inhibitor sirolimus. It is well known that pharmacogenetic profiles can influence efficacy and metabolization of drugs and interestingly, Yanagimachi et al*.* described that polymorphisms in the *CYP3A5* and *ABCB1* genes are associated with ciclosporin-related neurotoxicity [28] while Zhu et al*.* concluded that 28.3% of the difference in individual tacrolimus concentrations in patients with renal transplantation could be explained by CYP3A5*3 polymorphisms [29]. Rodríguez-Jiménez et al*.* looked at the effect of genetic polymorphisms of the *CYP3A5* and *ABCB1* genes on sirolimus-related side effects in renal transplant patients, but found no significant differences [30]. Clearance of sirolimus, however, was demonstrated to be lower in patients homozygous for the CYP3A5*3 allele [31]. There is still insufficient data to incorporate the polymorphisms of multiple genes in clinical utility models, but optimizing these models might lead to improved patient care.

In general, the precise pathogenic mechanisms that underlie neuroinflammation in disorders of immune tolerance are still elusive [32]. In particular, this also accounts for the CNS lymphocytic infiltrations in LRBA deficiency patients. The precise location of the initiating pathogenic event in the demyelination remains contentious. An ‘outside-in’ model, where autoreactive immune cells traffic in to the CNS and elicit autoimmune responses against myelin autoantigens, is placed against an ‘inside-out’ model, where pre-existing damage to oligodendrocytes and myelin prompts immune cell recruitment to the sites of injury, resulting in exaggerated inflammatory responses. Continued research is warranted to further clarify these pathogenetic mechanisms [33].

LRBA is a protein that is highly expressed in cochlear cells. Vogl et al*.* proposed that LRBA deficiency leads to degeneration of a fraction of stereocilia and therefore a reduction of cochlear action potentials upon hair bundle deflection. In turn, this results in a deficient cochlear amplification of sound and impaired sound encoding [25]. They referred to two patients with sensorineural hearing loss with no alternative explanation, initially described in [2] and [9]. We found an additional two patients [18, 23] with sensorineural hearing loss through literature review (see Table 1). Our two patients also had sensorineural hearing loss discovered as part of a screening audiometry for (pre-transplant) work-up, and both could not be sufficiently explained by ear infections, ototoxic medication or noise exposure. A genetic hearing loss gene panel was performed which revealed no alternative explanation. We conclude that their sensorineural hearing loss is most likely intrinsically caused by the LRBA deficiency. We therefore suggest the advice to perform annual screening of hearing in all patients with LRBA deficiency, as the hearing loss is relatively mild and slowly progressive, and may hence remain undetected clinically. As new treatments are likely to improve survival and quality of life, the sensorineural hearing loss could become more clinically relevant in the future [25].

In conclusion, we emphasize the importance of PID, such as LRBA deficiency, in the differential diagnosis in patients with inflammatory brain lesions. We strongly advocate for a more detailed description of CNS manifestations in patients with LRBA deficiency, when possible with MR imaging. This will aid clinical decision making with respect to both anti-infectious and anti-inflammatory therapy and in considering the indication for allo-HSCT. Sensorineural hearing loss is probably an underdiagnosed manifestation of LRBA deficiency, and we propose yearly hearing screening in all LRBA deficiency patients.

## Supplementary Information

**Additional file 1: Supplemental figure S1.** Diffusion-weighted imaging (DWI; a and b) demonstrating bilateral parieto-occipital cortical diffusion restriction. Apparent diffusion coefficient (ADC; c and d) map confirming diffusion restriction in these regions. Images in conjunction with those in Fig. 2 compatible with PRES. Images from patient P1.

**Additional file 2: Supplemental figure S2.** T2-FLAIR hyperintense contrast-enhanced lesions occurring widely spread in the supratentorial (a, b, c, d) and infratentorial (d) grey matter. Note as well the lesions in the lateral part of the right external globus pallidus (c, full arrow) and the left parahippocampal gyrus (d, dashed arrow). As in Fig. 2 and 3, the arachnoidal cyst in the left temporal region can also be observed (c, dashed arrow). Evolution after 6 months of treatment with abatacept can be seen on the follow-up images depicted with (ii), demonstrating global regression of the lesions and decreased contrast enhancement. Images from patient P1.

**Additional file 3: Supplemental figure S3.** Expansive lesion of the cervical spinal medulla from C3-C4 down to Th1-Th2. On the T1-weighted images (a and b) the patchy superficial contrast enhancement can be seen. Evolution after 6 months of treatment with abatacept can be observed on the follow-up images depicted with (ii), demonstrating reduced extent and decreased contrast enhancement. Images from patient P1.

**Additional file 4: Supplemental figure S4.** DWI images (a and b) demonstrating bilateral fronto-parieto-occipital cortical diffusion restriction. ADC map (c and d) confirming diffusion restriction in the cortex of these regions. Images in conjunction with those in Fig. 6 compatible with PRES. Images from patient P2.

## Data Availability

The datasets used and/or analysed during the current study are available from the corresponding author on reasonable request.

## Abbreviations

ADC: Apparent diffusion coefficient
ADEM: Acute disseminated encephalomyelitis
Ag: Antigen
AIHA: Auto-immune hemolytic anemia
Allo-HSCT: Allogeneic hematopoietic stem cell transplantation
APC: Antigen-presenting cell
CD: Cluster of differentiation
CNS: Central nervous system
CT: Computed tomography
CTLA-4: Cytotoxic T lymphocyte antigen 4
CVID: Common variable immunodeficiency disorders
DWI: Diffusion-weighted imaging
EEG: Electroencephalogram
FLAIR: Fluid-attenuated inversion recovery
GADA: Glutamic acid decarboxylase antibodies
GLILD: Granulomatous lymphocytic interstitial lung disease
ITP: Immune thrombocytopenic purpura
IVIG: Intravenous immunoglobulins
LETM: Longitudinally extensive transverse myelitis
LRBA: LPS-responsive beige-like anchor protein
MHC: Major histocompatibility complex
MRI: Magnetic resonance imaging
MS: Multiple sclerosis
PCR: Polymerase chain reaction
PID: Primary immunodeficiency
PRES: Posterior reversible encephalopathy syndrome
TCR: T cell receptor
WB: Western blot
WES: Whole exome sequencing
