# Supplementary figures and images for: Central nervous system manifestations of LRBA deficiency: case report of two siblings and literature review
Source: BMC Pediatr. 2023 Jul 13;23:353. doi: 10.1186/s12887-023-04182-z (PMC10339488; doi:10.1186/s12887-023-04182-z)

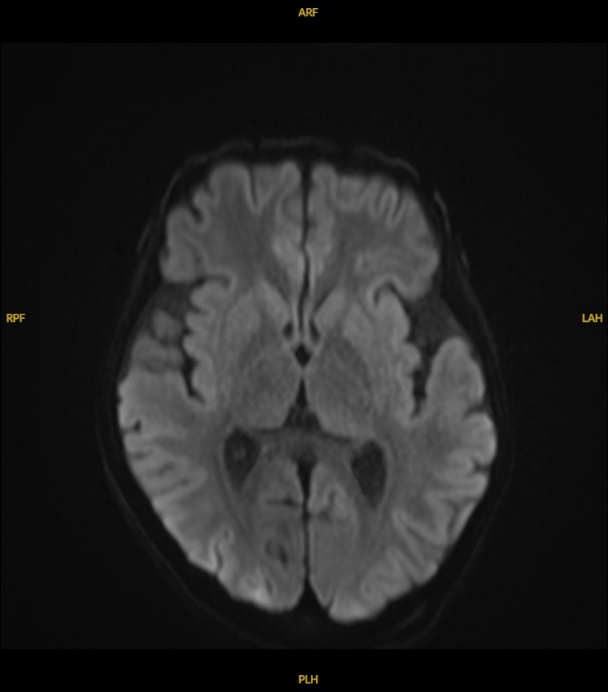

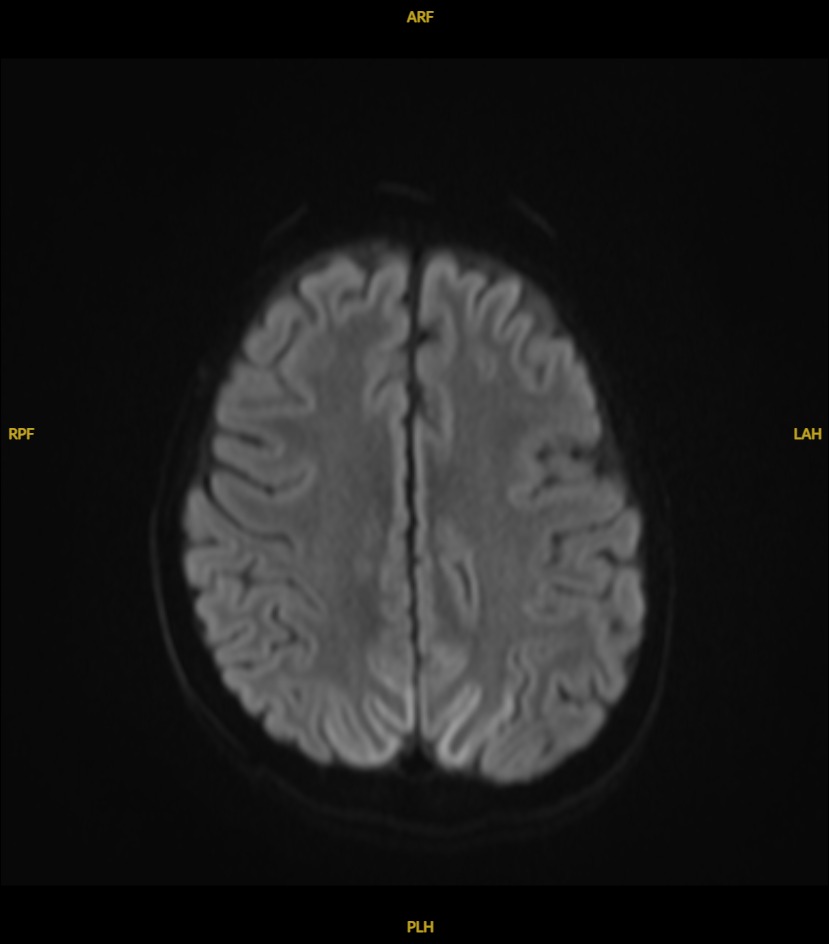


a

b


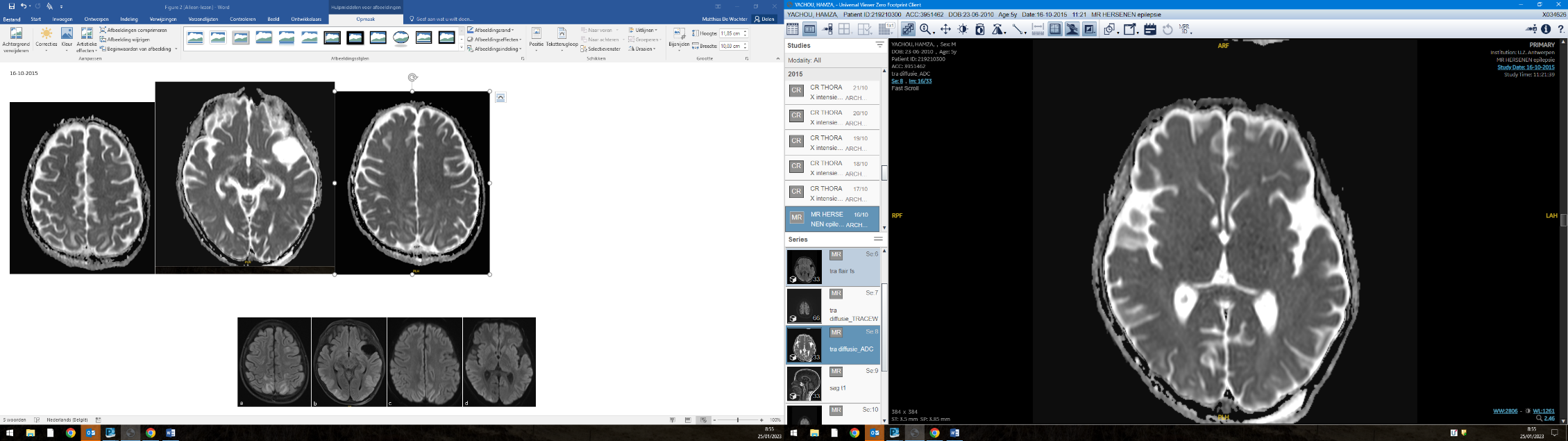

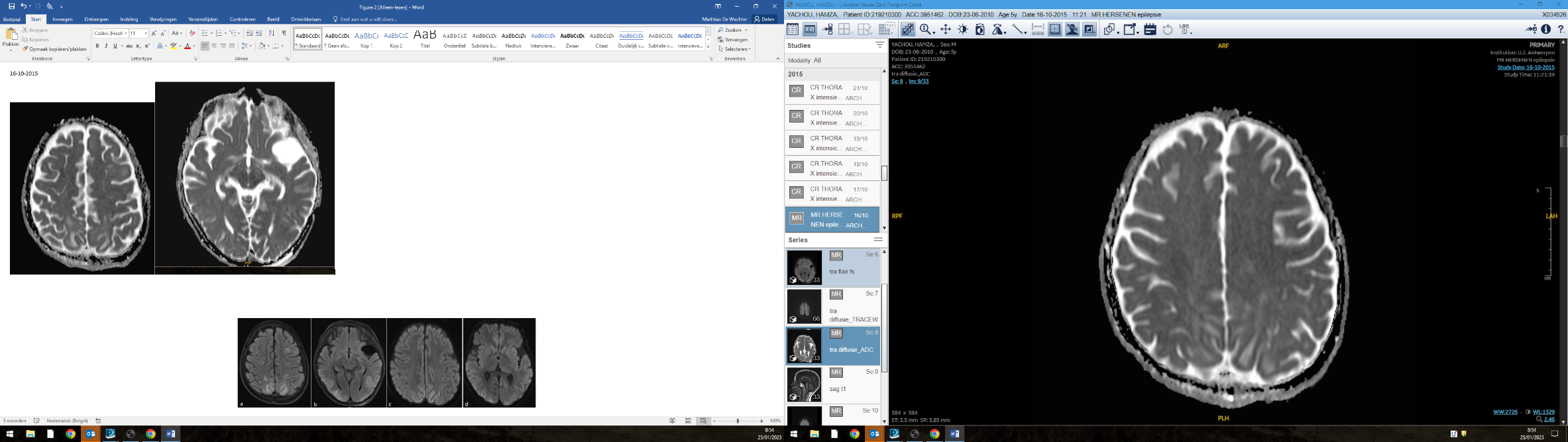


c

d

Supplement: Supplementary file 1 — Additional file 1: Supplemental figure S1. Diffusion-weighted imaging (DWI; a and b) demonstrating bilateral parieto-occipital cortical diffusion restriction. Apparent diffusion coefficient (ADC; c and d) map confirming diffusion restriction in these regions. Images in conjunction with those in Fig. 2 compatible with PRES. Images from patient P1. [file 12887_2023_4182_MOESM1_ESM.docx]

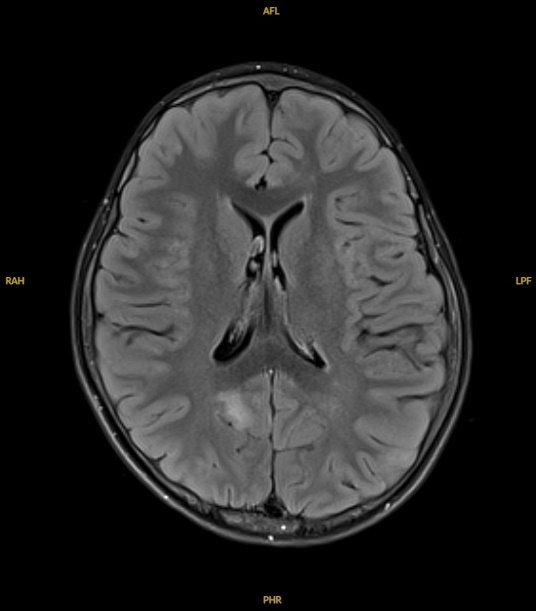


a(i)


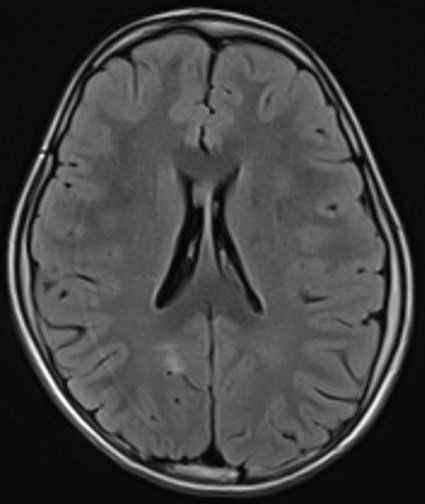


a(ii)


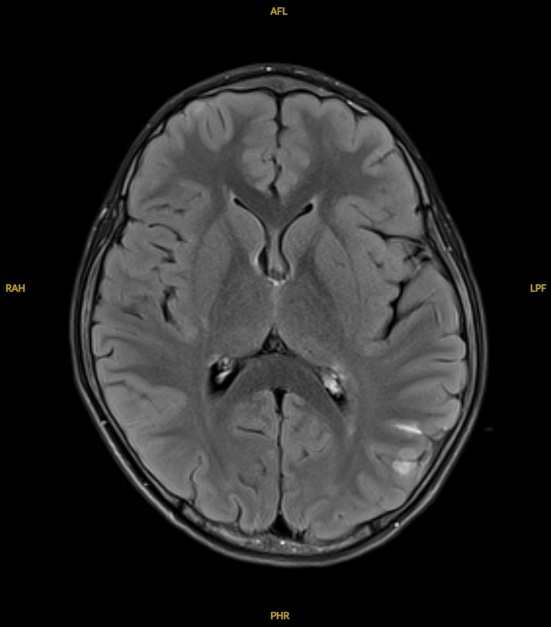


b(i)


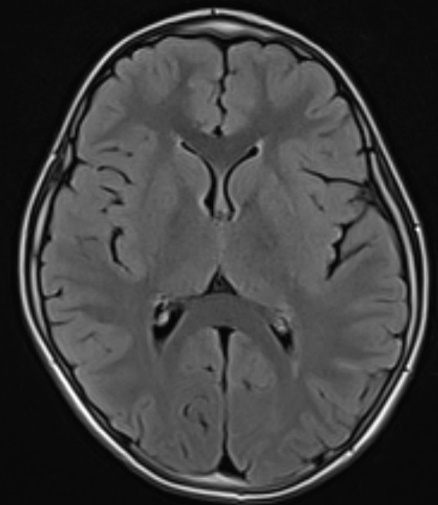


b(ii)


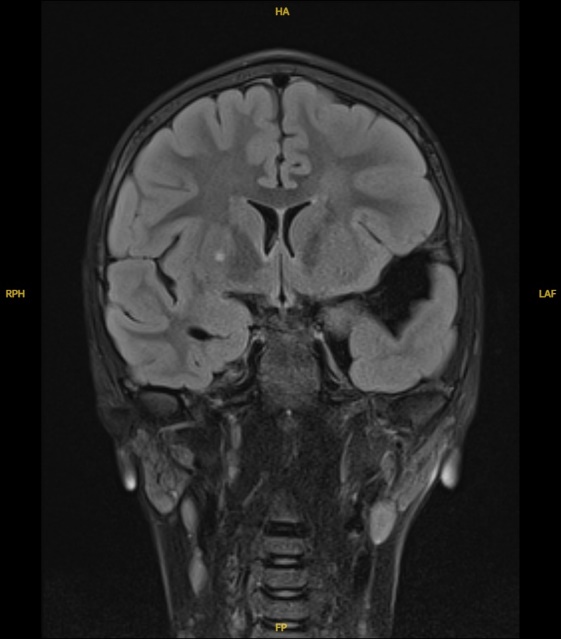


c


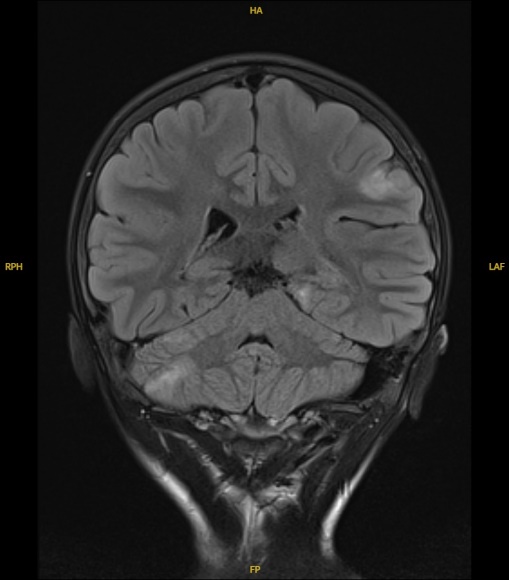


d

Supplement: Supplementary file 2 — Additional file 2: Supplemental figure S2. T2-FLAIR hyperintense contrast-enhanced lesions occurring widely spread in the supratentorial (a, b, c, d) and infratentorial (d) grey matter. Note as well the lesions in the lateral part of the right external globus pallidus (c, full arrow) and the left parahippocampal gyrus (d, dashed arrow). As in Fig. 2 and 3, the arachnoidal cyst in the left temporal region can also be observed (c, dashed arrow). Evolution after 6 months of treatment with abatacept can be seen on the follow-up images depicted with (ii), demonstrating global regression of the lesions and decreased contrast enhancement. Images from patient P1. [file 12887_2023_4182_MOESM2_ESM.docx]

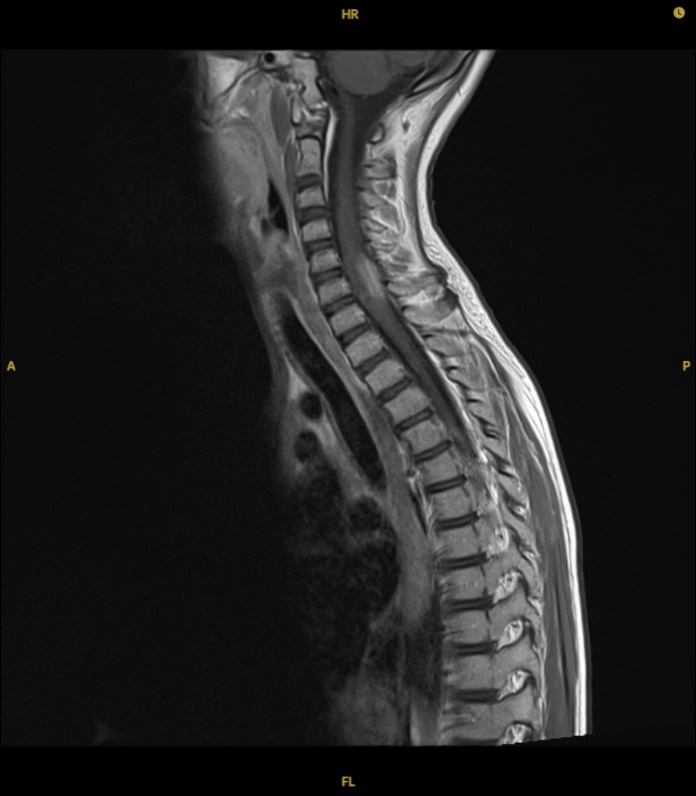

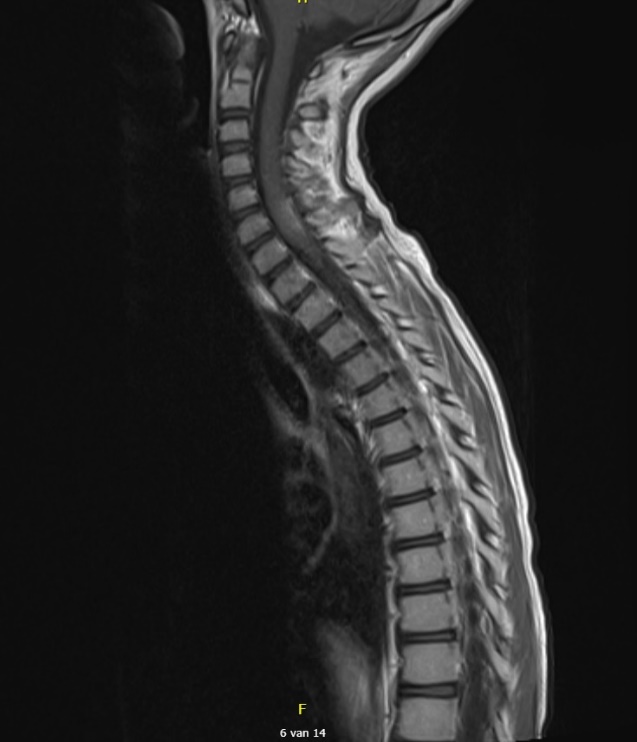


a(ii)

a(i)


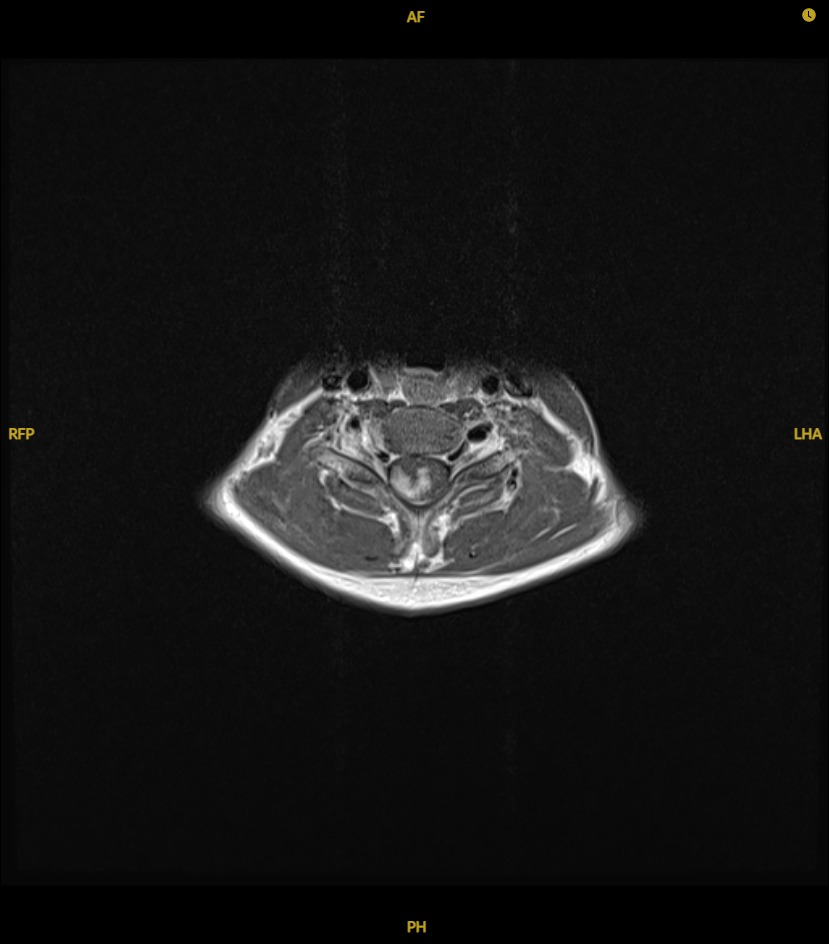

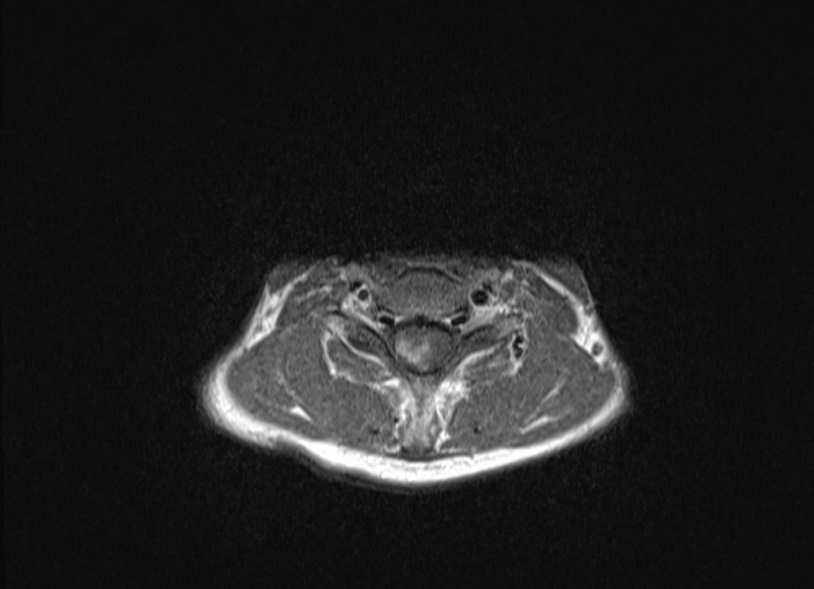


b(i)

b(ii)

Supplement: Supplementary file 3 — Additional file 3: Supplemental figure S3. Expansive lesion of the cervical spinal medulla from C3-C4 down to Th1-Th2. On the T1-weighted images (a and b) the patchy superficial contrast enhancement can be seen. Evolution after 6 months of treatment with abatacept can be observed on the follow-up images depicted with (ii), demonstrating reduced extent and decreased contrast enhancement. Images from patient P1. [file 12887_2023_4182_MOESM3_ESM.docx]

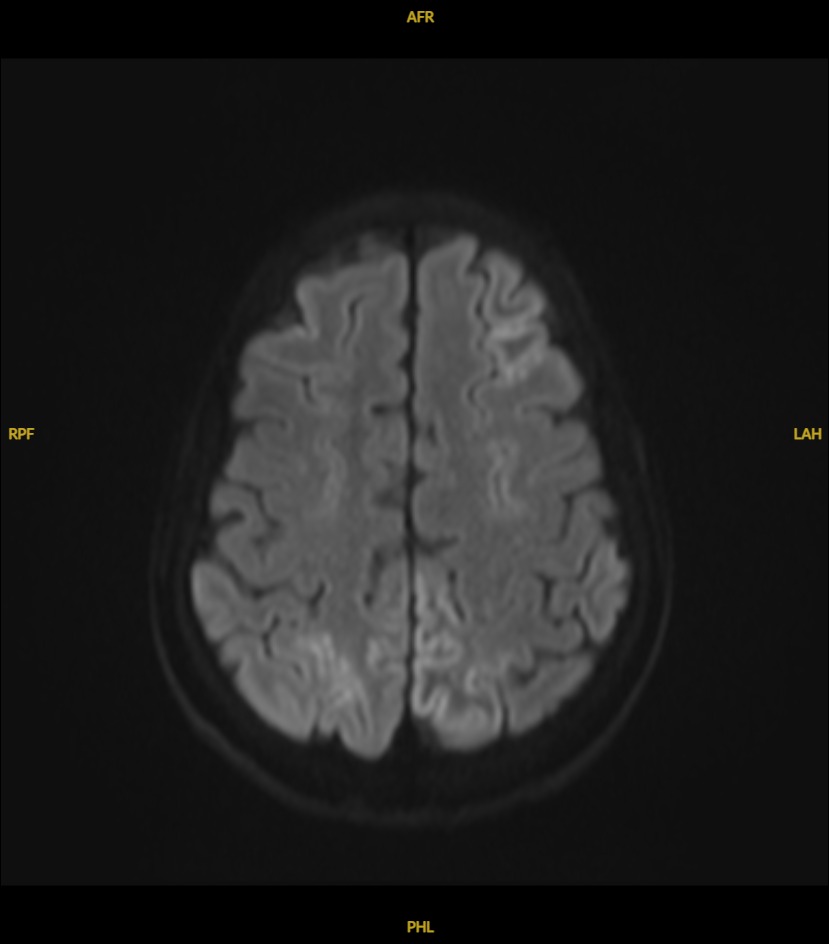

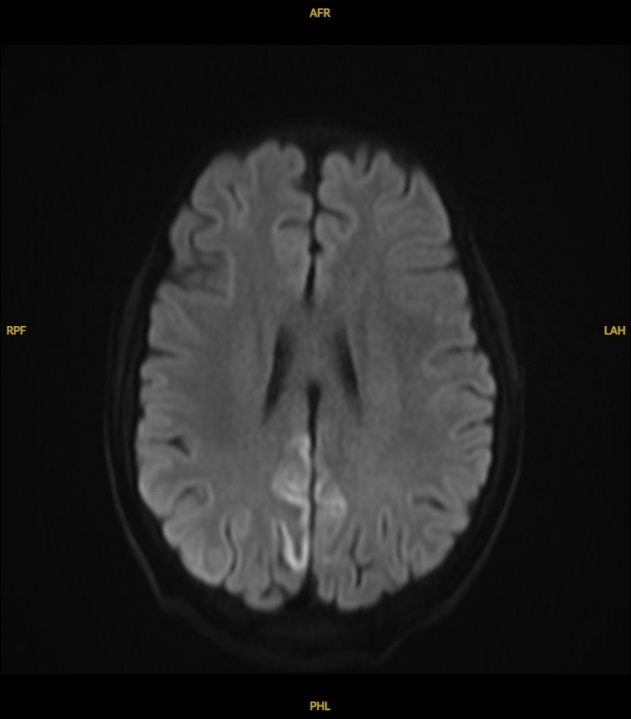


a

b


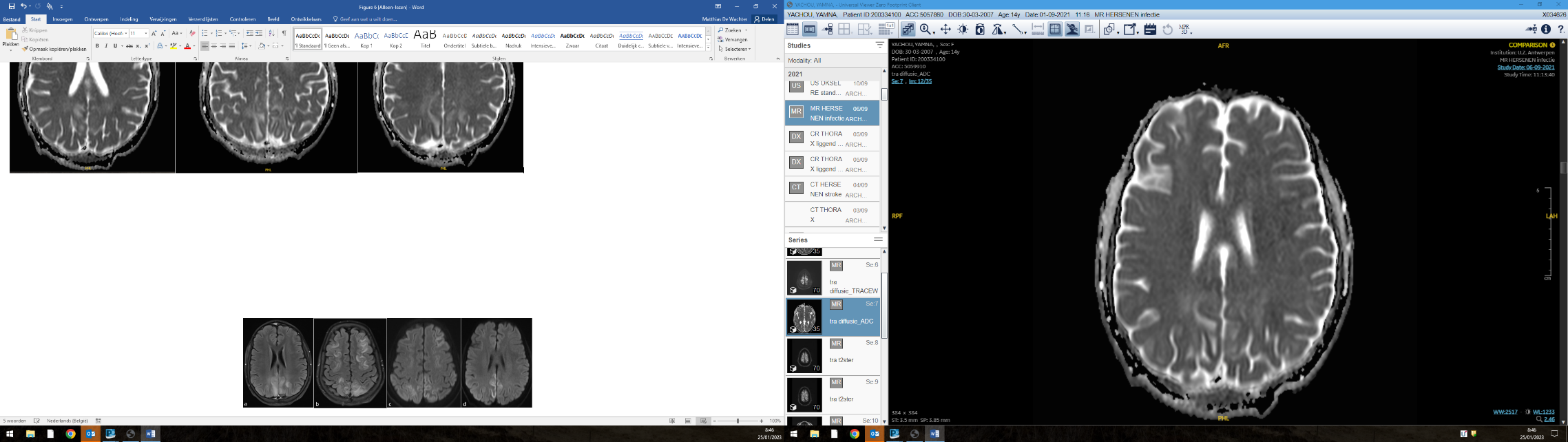

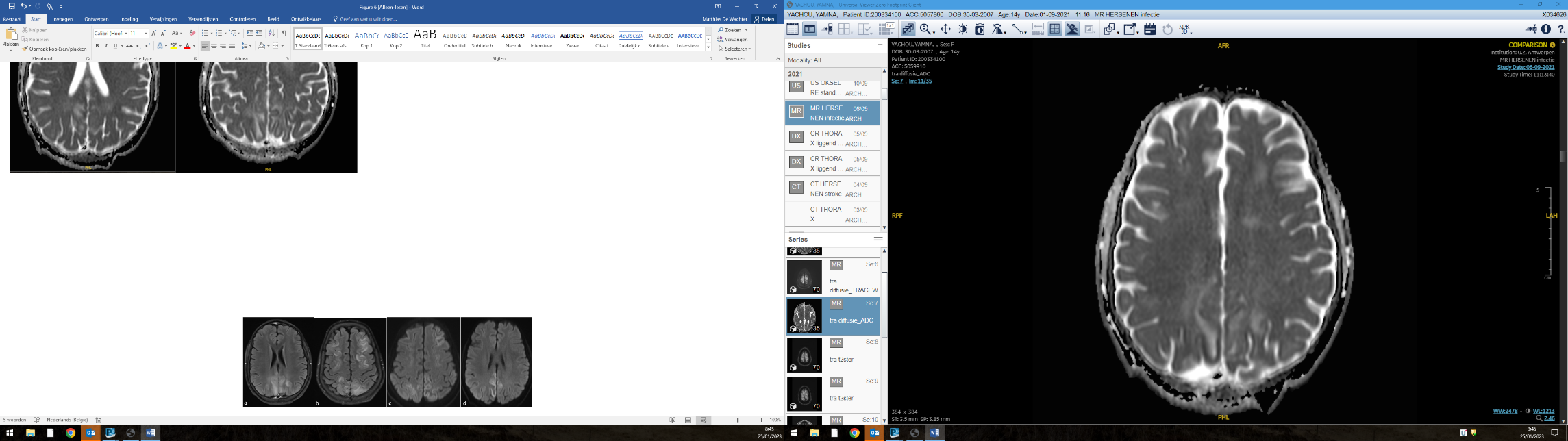


c

d

Supplement: Supplementary file 4 — Additional file 4: Supplemental figure S4. DWI images (a and b) demonstrating bilateral fronto-parieto-occipital cortical diffusion restriction. ADC map (c and d) confirming diffusion restriction in the cortex of these regions. Images in conjunction with those in Fig. 6 compatible with PRES. Images from patient P2. [file 12887_2023_4182_MOESM4_ESM.docx]
